# Tin-Based Perovskite Solar Cells: Structural Fundamentals, Material Engineering, and Prospects for Lead-Free Photovoltaics

**DOI:** 10.3390/nano16181138

**Published:** 2026-09-10

**Authors:** Bedelbek Nurbayev, Elena Dmitriyeva, Aigul Shongalova, Ainagul Kemelbekova

**Affiliations:** 1 Laboratory of Photovoltaic Phenomena and Devices, Institute of Physics and Technology, Satbayev University, Ibraimov Str., 11, Almaty 050013, Kazakhstan; b.nurbayev@satbayev.university (B.N.); y.dmitriyeva@satbayev.university (E.D.); a.shongalova@satbayev.university (A.S.); 2 Department of Materials Science, Nanotechnology and Engineering Physics, Satbayev University, Satbayev Str., 22a, Almaty 050013, Kazakhstan

**Keywords:** tin-based perovskites, lead-free perovskite solar cells, Sn^2+^ oxidation, tolerance factor, photovoltaic materials

## Abstract

Tin-based halide perovskites have emerged as one of the most promising classes of lead-free materials for next-generation photovoltaic technologies. Their favorable optoelectronic properties, narrow band gaps, and structural compatibility with the ABX_3_ perovskite framework position them as viable alternatives to conventional lead-based absorbers. This review summarizes the fundamental structural principles governing Sn-based perovskites, including the role of A-site cations, halide composition, tolerance factor, and octahedral distortion in determining phase stability and electronic structure. Recent advances in material engineering—such as additive-assisted crystallization, interface modification, precursor chemistry optimization, and two-dimensional/three-dimensional heterostructure formation—have significantly improved film quality, reduced defect densities, and enhanced device performance. Despite these achievements, challenges remain, particularly the spontaneous oxidation of Sn^2+^ to Sn^4+^, uncontrolled crystallization, and limited operational stability. Strategies aimed at stabilizing the Sn^2+^ oxidation state, suppressing self-doping, and improving charge transport are discussed in detail. The review also highlights the strategic importance of tin as a sustainable element for renewable energy technologies and provides an overview of global tin resources relevant to future photovoltaic deployment. Overall, tin-based perovskites represent a compelling pathway toward environmentally responsible and high-efficiency solar cells, with continued research expected to accelerate their transition from laboratory materials to commercially viable technologies.

## 1. Introduction

Humanity’s demand for energy continues to grow, and as the global population increases, the gap between required resources and their availability becomes increasingly evident [1]. Conventional methods of energy production are gradually reaching their limits, making the search for accessible, environmentally friendly, and renewable sources especially urgent. Against this backdrop, solar energy—particularly photovoltaic technologies—is viewed as one of the most promising approaches for meeting global energy needs. This has stimulated extensive research aimed at developing low-cost and highly efficient solar cells [2,3].

Although crystalline silicon still dominates the global solar cell market, thin-film technologies have also gained significant traction due to their low production cost and reduced material consumption [4]. Among the new generations of solar cells, perovskite-based devices have attracted particular attention. Within a short period, they have demonstrated a remarkable increase in efficiency and are now considered a potential alternative to existing photovoltaic technologies.

Perovskite solar cells are based on materials with an ABX_3_-type crystal structure, analogous to that of CaTiO_3_ [5,6]. In such compounds, A is a small organic or inorganic cation, B is a metal (most commonly Pb, Sn, or Ge), and X is a halide. The most extensively studied systems are hybrid organic–inorganic halide perovskites based on methylammonium and lead (MAPbX_3_), which have demonstrated outstanding optical and electronic properties. In 1958, Møller investigated the crystal structures and photoconductive properties of cesium lead halides, including CsPbCl_3_ and CsPbBr_3_, and identified their temperature-dependent perovskite structures [7]. This work initiated extensive investigations into the structural and electronic properties of halide perovskites. In 1978, Weber introduced hybrid organic–inorganic perovskites by elucidating their crystal structure and fundamental physicochemical characteristics, thereby laying the groundwork for the rapid development of modern perovskite optoelectronics and photonics [8]. The first report on organic–inorganic halide perovskites for photovoltaic applications appeared in 2009. In this work, Miyasaka and co-authors demonstrated a solar cell in which perovskite nanocrystals were deposited onto a mesoporous TiO_2_ scaffold and operated in conjunction with a liquid electrolyte. This study provided the first experimental evidence that hybrid halide perovskites could serve as efficient light-harvesting materials, marking the beginning of perovskite photovoltaics [9].

Despite their outstanding photovoltaic performance, lead-based perovskites face two major challenges: limited long-term stability and concerns regarding environmental safety [10]. The organic components of these materials are highly susceptible to degradation upon exposure to moisture, necessitating effective encapsulation strategies to ensure device durability. In addition, the presence of lead raises significant environmental and regulatory concerns, which may hinder the large-scale commercialization of perovskite photovoltaic technologies. To overcome these limitations, tin-based perovskites have emerged as one of the most promising lead-free alternatives, owing to the chemical similarity between tin and lead as Group 14 elements. Tin halide perovskites, such as CsSnI_3_ and MASnI_3_, possess narrow bandgaps and favorable optoelectronic properties, making them attractive candidates for the development of environmentally benign and potentially commercially viable perovskite solar cells [11,12].

Mitzi et al. were the first to synthesize a layered organic–inorganic halide perovskite exhibiting intense blue photoluminescence centered at approximately 460 nm, which was slightly blue-shifted relative to that of more three-dimensional perovskite analogues. Thermal stability measurements under an inert atmosphere revealed that the material remained stable up to approximately 290 °C, above which thermal decomposition was initiated [13].

In the early 2000s, Mitzi’s group continued to advance the field of layered perovskites by synthesizing new structures based on fluorophenylethylammonium and thoroughly investigating their optical properties and charge-transport behavior. These studies demonstrated that the organic component plays a crucial role in defining the material’s characteristics. Theoretical work further supported this conclusion, showing that variations in the size and spatial configuration of the organic cation can significantly influence the inorganic perovskite framework. Due to steric effects, the bandgap can change by nearly 1 eV, providing broad opportunities for the targeted tuning of the electronic properties of layered perovskites [14].

The development of tin-based perovskites has progressed substantially: from the earliest fundamental studies of their structure to the fabrication of high-quality thin films, detailed investigations of their optical and electronic properties, and the creation of strategies aimed at improving their stability and conductivity. Over time, it has become clear that controlling crystal chemistry, phase composition, and defect structure plays a crucial role in unlocking the potential of these materials. Owing to the absence of toxic lead and their favorable electronic characteristics, tin-based perovskites are regarded as an environmentally safer and highly promising alternative to traditional lead-containing perovskites, capable of delivering high efficiency and meeting the demands of future photovoltaic technologies [15,16,17,18].

## 2. Tin: Elemental Properties, Historical Use, and Strategic Significance

Tin (Sn, Z = 50) is a low-melting metal with an atomic mass of approximately 118.71. Under ambient conditions, it is stable and exhibits minimal corrosion due to the presence of a thin protective oxide film. Its key physical properties include a density of about 7280 g cm^−3^, a melting point of 231.9 °C, and a boiling point of 2620 °C. Tin possesses ten stable isotopes, with Sn-120 being the most abundant (~32.6%) [19]. Table 1 summarizes the key physical and chemical properties of elemental tin that are directly relevant to its behavior in photovoltaic materials and halide perovskite structures.

The metal occurs in two allotropic modifications: white tin (β-Sn), which has a tetragonal crystal structure, and gray tin (α-Sn), which adopts a diamond-like cubic lattice. Below the equilibrium transition temperature of 13.2 °C, metallic β-Sn becomes thermodynamically metastable with respect to brittle α-Sn. However, the β→α transformation is kinetically hindered and generally proceeds most rapidly at temperatures of approximately −30 to −40 °C, a phenomenon commonly known as tin pest. Tin forms soluble Sn^2+^ species (stannous compounds) and Sn^4+^ species (stannic compounds), and readily alloys with many metals (e.g., bronze, solders). Its principal compounds include SnO_2_ (an amphoteric dioxide), SnO, various chlorides (SnCl_2_, SnCl_4_), and organotin derivatives. At room temperature, tin does not react with air or water because it is protected by a thin layer of insoluble SnO_2_. Active oxidation begins only upon heating above 150 °C. The metal exhibits a metallic luster and relatively high thermal conductivity (66.8 W/m·K at 300 K). At room temperature, tin does not react with air or water because it is protected by a thin, insoluble SnO_2_ film. Noticeable oxidation begins only when the metal is heated above approximately 150 °C. Tin has a metallic luster and a relatively high thermal conductivity (66.8 W m^−1^ K^−1^ at 300 K). In its compounds, tin exhibits amphoteric behavior, forming both basic and acidic oxides and hydroxides, and typically occurs in two stable oxidation states, +2 and +4 [20,21,22,23,24].

Tin contains ten stable isotopes (112, 114–120, 122, 124) with atomic masses ranging from 111.90 to 123.90 [25]. This broad isotopic range results in a relatively high average atomic mass (118.71). Above 13.2 °C, the thermodynamically stable allotrope of tin is metallic β-Sn, which has a body-centred tetragonal crystal structure, whereas below 13.2 °C, the diamond-cubic α-Sn phase is thermodynamically favoured. Under high pressure, additional γ- and σ-phases have been identified. The Debye temperatures are about 170 K for β-Sn and 110 K for α-Sn. Tin is largely inert under ambient conditions. It dissolves in hot acids, forming SnCl_2_ or SnCl_4_ depending on the reaction conditions. Oxidation generally produces SnO_2_, although SnO, hydroxides, and various organotin compounds (e.g., PVC stabilizers) are also known [26].

Tin forms eutectic and wide-range alloys with many metals, including lead, copper, silver, and bismuth. The Sn–Pb system contains a single eutectic at 61.9% Sn (melting point 183.3 °C) [27]. At other compositions, the crystallization interval is nonzero. Modern lead-free solders, such as Sn–Ag–Cu alloys, are technologically important, though their detailed characteristics are beyond the scope of this summary [28]. At room temperature, tin is passivated by a stable oxide film. When heated above 200–300 °C, it gradually oxidizes to SnO_2_. Tin coatings provide partial protection to underlying metals such as steel, helping to prevent corrosion and reducing the risk of lead contamination in food-related applications.

**Figure 1:**
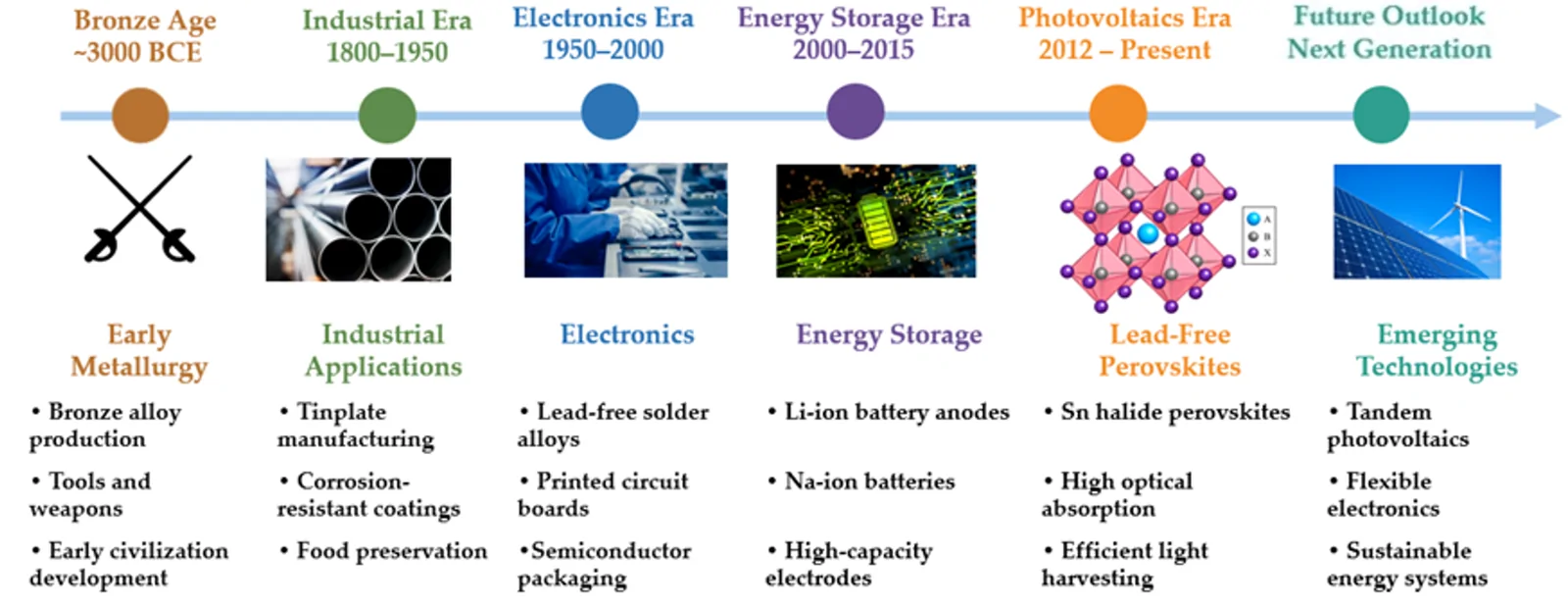
Chronological evolution of tin-based materials and technologies.

Tin is one of the earliest metals exploited by humans, with documented use dating back to approximately 3500 BCE. The discovery of bronze, produced by alloying tin with copper around 2500 BCE, initiated the Bronze Age and established tin as a material of major technological significance [29]. Over subsequent centuries, its applications expanded from bronze production to tinplate manufacturing, food preservation, and industrial coatings, ultimately evolving toward modern electronic, energy-storage, and photovoltaic technologies [30,31,32,33,34,35,36]. Figure 1 illustrates the chronological evolution of tin-based materials and technologies, highlighting their development from ancient metallurgy to modern and emerging applications in sustainable energy. The timeline summarizes the major milestones, spanning the Bronze Age and the Industrial Revolution to the advent of electronics, energy storage technologies, and contemporary tin-based perovskite solar cells.

### 2.1. Global Tin Resources and Production

According to recent assessments of global tin reserves, the distribution of economically recoverable resources remains highly uneven, with a strong concentration in a limited number of countries. As shown in the reserve structure, Indonesia holds the largest share of global tin reserves, accounting for 22% of the total. China follows with 19%, reflecting its long-standing role as both a major producer and consumer of refined tin. Brazil (12%) and Myanmar (11%) also represent significant reserve holders, underscoring the importance of Southeast Asia and South America in the global tin supply chain.

Australia contributes 10% of global reserves, while Russia and Bolivia account for 8% and 7%, respectively. These countries maintain strategic positions in the tin market due to their established mining sectors and export capacities. The remaining reserves are distributed among several nations with smaller shares, including Peru (3%), the Democratic Republic of Congo (2%), and Vietnam (1%). Collectively, “other countries” represent 5%, indicating that tin reserves outside the major producers are relatively limited.

The concentration of tin reserves in Indonesia, China, and Brazil highlights the geopolitical sensitivity of the tin market. These countries collectively control more than half of global reserves, making their production policies, environmental regulations, and export strategies critical factors influencing global supply stability. For countries with smaller reserve bases, such as Peru or Vietnam, tin production remains economically important but does not significantly affect global market balance.

**Figure 2:**
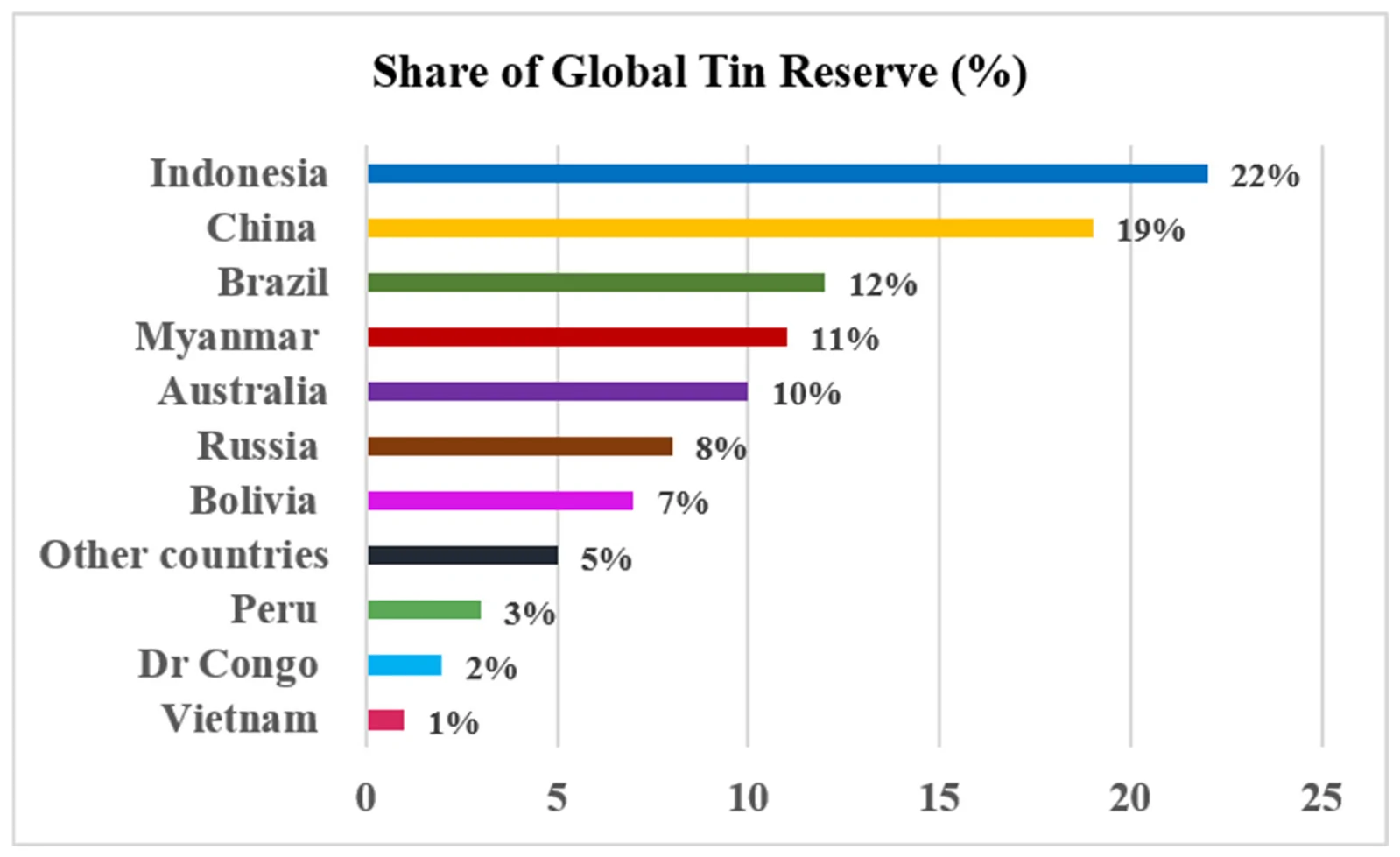
Distribution of tin reserves by country.

Figure 2 provides a visual representation of how global tin reserves are distributed among major producing countries, while Table 2 summarizes the corresponding production levels and reserve estimates for 2024 and 2025.

Global tin reserves are estimated to exceed 6 million metric tons, with the largest confirmed deposits located in Indonesia (1.4 million t), China (1.2 million t), Brazil and Burma (each 700,000 t), and Australia (570,000 t). Significant reserve bases are also present in Bolivia (400,000 t) and Russia (460,000 t). In contrast, several producing countries—including Laos, Malaysia, Nigeria, and Rwanda—do not report official reserve figures, complicating comprehensive global assessments.

Table 2 summarizes tin extraction volumes (in metric tons) for major producing countries and provides the most recent estimates of their mineral reserves. All numerical values are derived from national geological surveys and international analytical sources, including the USGS (2025) [37].

### 2.2. Strategic Importance of Tin for Renewable Energy

Perovskite solar cells (PSCs) have emerged as one of the most transformative photovoltaic technologies due to their exceptional power conversion efficiency (PCE), low-cost fabrication, and compatibility with scalable solution-processing techniques [39,40]. Since their initial demonstration with a power conversion efficiency of 3.8%, single-junction perovskite solar cells have undergone rapid development, achieving a certified efficiency of 27.2% and thereby approaching 28% [41,42,43]. This remarkable progress has been attributed to the unique optoelectronic properties of halide perovskites, including high absorption coefficients, long carrier diffusion lengths, low exciton binding energies, and tunable band gaps. In addition to photovoltaic applications, metal halide perovskites have demonstrated significant potential in light-emitting diodes, photodetectors, lasers, and photocatalytic systems, making them versatile materials for next-generation renewable energy technologies [44,45,46]. Despite their outstanding photovoltaic performance, lead-based perovskites face substantial barriers to large-scale commercialization. The primary concern is the presence of toxic Pb^2+^ ions, which pose environmental and health risks during material synthesis, device operation, disposal, and recycling. Under humid conditions, degradation of lead perovskites can result in the release of soluble lead compounds capable of contaminating soil and groundwater. Furthermore, compliance with increasingly stringent environmental regulations, including restrictions on hazardous substances, presents additional challenges for commercial deployment. Another limitation is the insufficient long-term operational stability of lead-based PSCs, as exposure to moisture, oxygen, heat, and ultraviolet irradiation accelerates degradation processes and reduces device lifetime. These issues have stimulated intensive research into environmentally benign alternatives capable of maintaining comparable optoelectronic performance. Among all potential lead substitutes, tin has attracted the greatest attention because of its chemical similarity to lead and its ability to preserve the three-dimensional ABX_3_ perovskite crystal structure. As a group 14 element, Sn^2+^ possesses an electronic configuration comparable to Pb^2+^, allowing the formation of structurally compatible perovskites while significantly reducing environmental toxicity. Tin-based iodide perovskites typically exhibit narrow bandgaps of approximately 1.1–1.4 eV, depending on their composition and crystal structure. This range is close to the Shockley–Queisser optimum for single-junction solar cells [47,48,49]. These characteristics make tin-based absorbers particularly attractive for high-efficiency tandem photovoltaics and near-infrared light harvesting. Beyond environmental considerations, the transition from lead to tin aligns with global sustainability strategies, circular economy principles, and international efforts to develop safer materials for renewable energy technologies. Consequently, tin has become a strategically important element for the next generation of environmentally responsible perovskite photovoltaics.

Tin-based perovskites possess several intrinsic advantages that make them promising candidates for replacing lead-containing materials [50]. Their narrower band gap enables broader solar spectrum absorption and potentially higher theoretical photocurrent densities. In addition, Sn-based perovskites exhibit high charge-carrier mobility, relatively low exciton binding energies, and strong optical absorption comparable to those of lead analogues [51]. The absence of highly toxic lead significantly improves their environmental compatibility and facilitates future commercialization under increasingly strict environmental regulations. Recent advances in defect engineering, additive-assisted crystallization, interface modification, two-dimensional/three-dimensional heterostructures, and compositional engineering have substantially improved film quality, reduced defect densities, and enhanced photovoltaic performance. These developments indicate that tin-based perovskites are evolving from laboratory-scale materials toward viable candidates for sustainable photovoltaic technologies.

Despite significant progress, several critical challenges continue to limit the practical application of tin-based perovskite solar cells. The spontaneous oxidation of Sn^2+^ to Sn^4+^ remains the principal obstacle, leading to high background carrier concentrations, increased defect densities, accelerated non-radiative recombination, and rapid material degradation. Additional issues include uncontrolled crystallization, poor film uniformity, interface instability, and relatively low open-circuit voltages compared with lead-based counterparts. Future research should focus on stabilizing the Sn^2+^ oxidation state through advanced precursor chemistry, reducing defect formation, optimizing charge transport layers, and developing encapsulation strategies that improve long-term operational stability. Moreover, integrating machine learning, high-throughput materials screening, and computational materials design is expected to accelerate the discovery of novel tin-based compositions with enhanced efficiency and durability. Continued progress in these areas will determine whether tin-based perovskites can transition from promising laboratory materials to commercially viable photovoltaic technologies.

## 3. Fundamentals and Classification of Tin-Based Perovskites

Hybrid organic–inorganic and all-inorganic halide perovskites are commonly represented by the general formula ABX_3_. The A site is occupied by a relatively large monovalent cation, such as methylammonium (MA^+^), formamidinium (FA^+^), or Cs^+^; the B site contains a smaller divalent metal cation, typically Sn^2+^ or Pb^2+^; and the X site is occupied by a halide anion, including I^−^, Br^−^, or Cl^−^. In the ideal cubic structure with the ($Pm\bar{3}m$) space group, each B-site cation is coordinated by six halide anions, forming a [BX_6_] octahedron. The octahedra are connected through shared vertices to produce a continuous three-dimensional framework, with the A-site cations located within the resulting cuboctahedral cavities.

The formation and stability of this framework depend on the geometric compatibility of the constituent ions. Changes in ionic radii can modify the B–X bond lengths, B–X–B bond angles, degree of octahedral tilting, and lattice symmetry. These structural parameters govern orbital overlap within the inorganic framework and consequently affect the band structure, optical absorption, charge-carrier transport, defect energetics, and phase stability. Therefore, only suitable combinations of A-, B-, and X-site ions can support a stable three-dimensional perovskite lattice.

**Figure 3:**
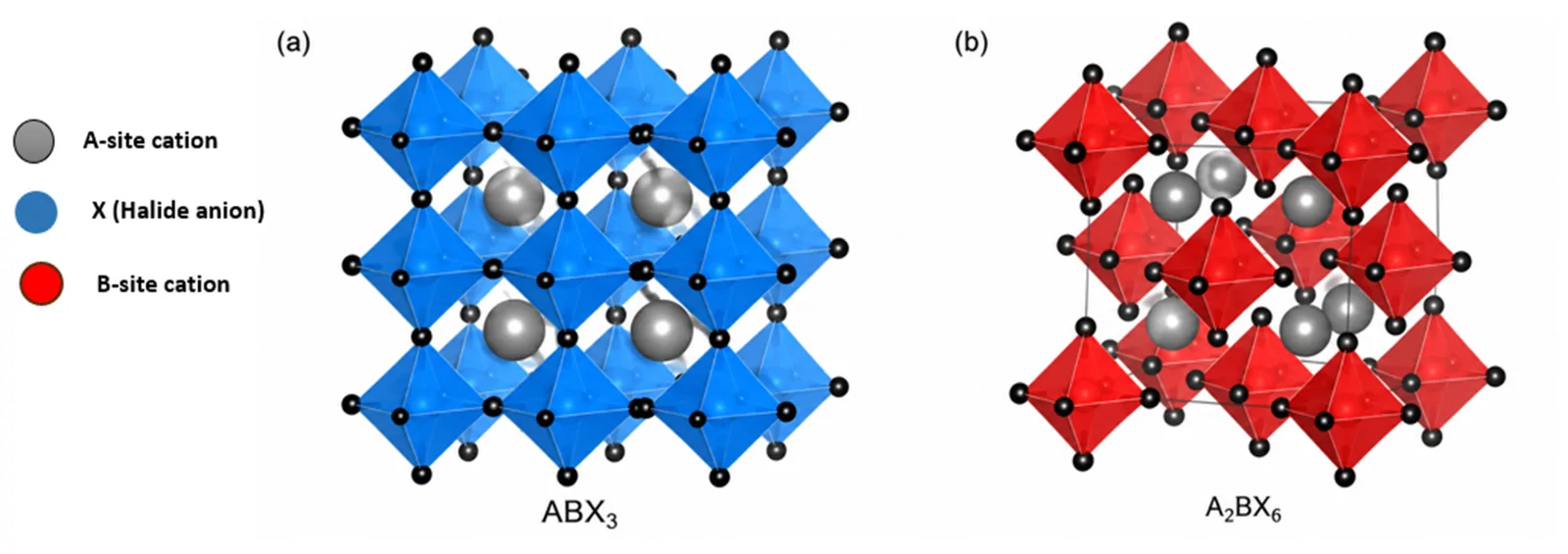
Crystal structures of ABX3 (space group = $Pm\bar{3}m$) (**a**) and A2BX6 (space group = $Fm\bar{3}m$) (**b**). Reproduced from Ref. [52].

Vacancy-ordered halide perovskites with the general formula A_2_BX_6_ represent a structurally distinct derivative of the ABX_3_ framework. In tin-based A_2_BX_6_ compounds, the B site is occupied by Sn^4+^ rather than Sn^2+^, ensuring charge neutrality. Their structure can be described as an ABX_3_-derived lattice in which half of the B-site positions are systematically vacant. Consequently, the [BX_6_] octahedra are isolated from one another instead of being connected through shared vertices. The absence of direct octahedral connectivity modifies orbital interactions and band dispersion, generally leading to more localized electronic states and different charge-transport and defect characteristics compared with three-dimensional ABX_3_ perovskites. Figure 3 illustrates the ideal cubic ABX_3_ structure and its vacancy-ordered A_2_BX_6_ derivative [52].

Two empirical geometric descriptors are commonly used to evaluate the compatibility of ABX_3_ compositions: the Goldschmidt tolerance factor (t) and the octahedral factor (μ). The tolerance factor is defined as [53,54].

$$t=\frac{R_{a}+R_{x}}{\sqrt{2}(R_{b}+R_{x})}$$

where $R_{a}$, $R_{b}$, and $R_{x}$ denote the effective ionic radii of the A-site cation, B-site cation, and halide anion, respectively. This parameter describes the fit of the A-site cation within the cavity formed by the corner-sharing BX_6_ octahedra. Three-dimensional halide perovskites are frequently observed when (t) lies approximately between 0.8 and 1.0. Lower values are generally associated with stronger octahedral tilting and lower-symmetry structures, whereas values approaching unity tend to favour a more symmetric, cubic-like framework. The second descriptor, the octahedral factor, is defined as

$$\mu =\frac{R_{B}}{R_{X}}.$$

It describes the geometric compatibility of the B-site cation with the octahedral coordination environment formed by the surrounding X-site anions. Thus, (t) primarily evaluates the accommodation of the A-site cation within the perovskite cavity, whereas (µ) evaluates the ability of the B-site cation to form stable BX_6_ octahedra. These parameters provide useful initial structural guidelines but should not be treated as definitive predictors of phase stability because they do not fully account for temperature, pressure, chemical bonding, lattice dynamics, or configurational effects [53,54].

The optical and electronic properties of tin-based perovskites are closely linked to the geometry of their SnX_6_ framework. Variations in the A-site cation and halide composition modify the Sn–X bond lengths, Sn–X–Sn bond angles, octahedral tilting, and lattice symmetry. These structural changes can influence orbital overlap, band dispersion, bandgap, carrier effective masses, defect formation, and charge-transport properties. Accordingly, the tolerance and octahedral factors should be considered together with experimentally determined structural parameters when evaluating Sn-based perovskites [55].

Among commonly used A-site cations, the relatively large FA^+^ cation (effective radius ≈ 253 pm) generally reduces octahedral tilting in FASnI_3_ and favours a quasi-cubic photoactive structure at room temperature. The smaller MA^+^ cation (≈217 pm) produces a different degree of lattice distortion, and MASnI_3_ undergoes a tetragonal-to-orthorhombic phase transition at low temperature, accompanied by changes in lattice strain and bandgap. The still smaller inorganic Cs^+^ cation (≈167 pm) promotes stronger octahedral tilting in CsSnI_3_ and contributes to its complex temperature-dependent phase behaviour. Under ambient conditions, the photoactive black perovskite phase of CsSnI_3_ is also susceptible to transformation into a yellow non-perovskite phase. These examples demonstrate that the A-site cation affects optoelectronic properties indirectly by controlling octahedral geometry, lattice symmetry, and phase stability. Moreover, the cubic-to-orthorhombic phase transition near 300 K increases octahedral tilting and modifies orbital overlap, which may reduce band dispersion, increase carrier effective masses, and hinder charge transport [56].

Besides A-site cation engineering, halide composition also plays a critical role in regulating the structural and optoelectronic properties of Sn-based perovskites. Partial substitution of iodide with bromide or chloride enables effective bandgap tuning while simultaneously influencing phase stability and lattice distortion. Overall, the interplay between cation size, halide composition, tolerance factor, and octahedral distortion governs the phase evolution and optoelectronic performance of Sn-based perovskites, emphasizing the importance of rational compositional and structural engineering for achieving highly efficient and stable lead-free perovskite solar cells.

Tin-based halide perovskites face several intrinsic and extrinsic challenges that critically limit their photovoltaic performance and stability, which can be systematically understood through a problem–mechanism–strategy framework. The facile oxidation of Sn^2+^ to Sn^4+^ introduces deep-level defects and accelerates degradation, necessitating reducing additives, inert processing, and encapsulation to stabilize the oxidation state. Oxidized Sn^4+^ species also induce p-type self-doping, elevating hole concentrations and lowering open-circuit voltage, which can be mitigated by defect passivation, compositional engineering, and controlled doping. Rapid nucleation and growth often lead to uncontrolled crystallization, poor film coverage, and high trap densities, requiring solvent engineering, nucleation-layer assisted growth, and 2D/3D heterostructures to regulate film formation. Weak lattice stability promotes high defect density through vacancies and interstitials, creating non-radiative recombination centers, which can be suppressed by halide additives, fullerene derivatives, and interface engineering. Large *V*oc losses arise from defect-assisted recombination and high background doping, highlighting the need for optimized band alignment, trap reduction, and interfacial modification. Finally, poor operational stability due to environmental sensitivity and phase transitions demands encapsulation, mixed cation/halide compositions, and robust charge-transport layers. Collectively, these strategies demonstrate that overcoming oxidation, self-doping, crystallization control, defect passivation, voltage loss, and stability issues requires an integrated approach combining compositional design, interface optimization, and protective device architectures to unlock the full potential of lead-free tin perovskite photovoltaics.

### 3.1. Methylammonium Tin Halides (MASnX_3_)

MASnI_3_ is a direct-bandgap semiconductor in which the valence- and conduction-band edges occur at the same point in reciprocal space, enabling efficient photon absorption across the visible and near-infrared regions of the solar spectrum. Both MASnI_3_ and CsSnI_3_ exhibit bandgap values in the range of approximately 1.1–1.4 eV, making them attractive absorber materials for high-performance photovoltaic devices due to their strong optical absorption [57]. In contrast, theoretical studies predict that the hexagonal polymorph of MASnI_3_ possesses a fundamentally different electronic structure, characterized by an indirect bandgap of approximately 2.3 eV. Such a wider bandgap shifts the absorption edge toward higher photon energies, extending into the ultraviolet region.

The electronic structure of MASnI_3_ is governed primarily by the SnI_6_ octahedral framework. The upper valence band is mainly derived from antibonding interactions between Sn 5s and I 5p orbitals, whereas the conduction band minimum is dominated by Sn 5p states. Consequently, structural variations within the octahedra, including changes in the Sn–I bond length and Sn–I–Sn bond angle, have a direct impact on the bandgap energy. Although the A-site cation does not directly contribute to the frontier electronic states, it can significantly modify the electronic structure by inducing octahedral tilting, lattice distortion, and symmetry changes. In certain cases, the organic A-site cation may have a stronger influence on the electronic structure than previously assumed, because the p orbitals of its nitrogen and carbon atoms can partially contribute to the upper edge of the valence band, particularly under substantial lattice distortion [58].

Therefore, the bandgap of tin halide perovskites is governed not only by the chemical nature of the B- and X-site ions but also by subtle structural effects associated with the size, geometry, and dynamic behavior of the A-site cation. As a result, the optoelectronic properties of these materials are highly sensitive to phase transitions, crystal symmetry, and local lattice distortions, all of which must be carefully considered in the design and optimization of perovskite solar cells and other optoelectronic devices.

MASnI_3_ exhibits a sequence of temperature-dependent structural transitions involving pseudocubic, tetragonal, and orthorhombic phases. As summarized in Table 3, cooling below approximately 275 K induces the transition from the pseudocubic α phase to the tetragonal β phase, whereas a further decrease in temperature to approximately 108 K produces the orthorhombic γ phase. These transitions are associated with progressive SnI_6_ octahedral tilting, reduced lattice symmetry, and increased orientational ordering of the MA^+^ cations.

At room temperature, MASnI_3_ adopts a dynamically averaged pseudocubic α structure. Upon cooling below approximately 275 K, increased tilting of the SnI_6_ octahedra lowers the symmetry and produces the tetragonal β phase. The subsequent transition to the orthorhombic γ phase occurs at approximately 108 K during cooling and at approximately 114 K during heating, indicating a small thermal hysteresis. Although this low-temperature transition causes only a limited change in the band-edge energy, it substantially influences defect-related optoelectronic properties. In particular, the orthorhombic phase exhibits reduced photoluminescence linewidth, lower free-hole concentration, and an increased charge-carrier lifetime, suggesting a change in the energetic position or ionization behavior of defects such as Sn vacancies.

A variety of methods have been proposed for obtaining high-quality tin-based perovskite films [60]. For example, a chemical approach has been developed for producing high-quality methylammonium tin iodide (MASnI_3_) perovskite films. In the first step, a hydrazinium tin iodide (HASnI_3_) perovskite film is deposited from a solution containing hydrazinium iodide (HAI) and tin iodide (SnI_2_), after which it is converted into MASnI_3_ through a cation-exchange process. This two-step procedure enables the formation of a dense and uniform MASnI_3_ film with large grains and a high degree of crystallinity. Harmful oxidation is effectively suppressed by hydrazine released from the film during the transformation. Using MASnI_3_ as the light-absorbing layer, mesoporous perovskite solar cells were fabricated and achieved a power conversion efficiency (PCE) of 7.13% [61].

In addition to experimental investigations, numerical device and optical simulations have been employed to evaluate the theoretical performance potential of MASnI_3_-based solar cells. Ivriq et al. reported a simulation study in which the incorporation of a CH_3_NH_3_SnI_3_ absorber layer into the modeled device architecture resulted in a predicted PCE of 26.89%. The simulated introduction and optimization of Au and Ag metallic nanoparticles within the MASnI_3_ layer increased the calculated PCE to 29.52% by enhancing light absorption. More stable dielectric–metal–dielectric nanoparticles, including TiO_2_@Ag@TiO_2_ and SiO_2_@Ag@SiO_2_, were subsequently evaluated in the optical model and were predicted to improve the short-circuit current density by approximately 40%. Under the optimized simulation conditions, the modeled device exhibited a predicted *V*oc of 1.01 *V*, *J*sc of 35.17 mA cm^−2^, FF of 84.16%, and PCE of 30.18%. These values represent theoretical predictions obtained through device and optical modeling and should not be interpreted as experimentally measured efficiencies. Nevertheless, the results indicate the potential of plasmonic nanostructures as a strategy for improving light management in MASnI_3_-based solar cells, subject to future experimental validation [62].

### 3.2. Formamidinium Tin Halides (FASnI_3_)

FASnI_3_ belongs to three-dimensional perovskite structures and is characterized by a direct absorption edge with a transition energy of about 1.41 eV. The electronic structure of FASnX_3_ perovskites can be deliberately tuned through isoelectronic substitution of halide anions, enabling controlled modification of the bandgap. In particular, partial replacement of iodide with bromide increases the interband transition energy: for FASnI_2_Br it is approximately 1.68 eV, while for the fully bromide analogue FASnBr_3_ it reaches 2.4 eV [63]. Mitzi and Liang were the first to synthesize and characterize the cubic FASnI_3_ perovskite in 1997. Owing to its improved air stability, FASnI_3_ has recently become the material of choice in most high-performance Sn-based perovskite solar cells [64,65].

FASnI_3_ undergoes a sequence of temperature-induced structural transitions upon cooling. As summarized in Table 4, the material transforms from the cubic α phase into the tetragonal β phase and subsequently into the low-temperature orthorhombic γ phase. These transformations are accompanied by progressive SnI_6_ octahedral tilting and increasing orientational ordering of the FA^+^ cations.

At room temperature, FASnI_3_ adopts a cubic $Pm\bar{3}m$ structure characterized by dynamic orientational disorder of the FA^+^ cations. Upon cooling through approximately 250–225 K, in-plane rotations of the SnI_6_ octahedra reduce the lattice symmetry and produce the tetragonal *P4/mbm* phase. A further transition occurs between approximately 150 and 125 K, resulting in the orthorhombic *Pnma* structure. The increasing octahedral tilting and cation ordering modify the Sn–I–Sn bond angles and orbital overlap and may therefore influence the bandgap and charge-carrier behavior [66].

In the study conducted by Meng et al. [67], a strategy was proposed to slow down the crystallization kinetics of the FASnI_3_ perovskite through the introduction of hydrogen bonding. The incorporation of poly(vinyl alcohol) (PVA) leads to the formation of O–H…I^−^ hydrogen-bond interactions between PVA and FASnI_3_, which promotes the creation of additional nucleation sites, moderates crystal growth, guides crystal orientation, reduces trap-state density, and suppresses iodide-ion migration. With the incorporation of PVA, FASnI_3_–PVA solar cells achieved a power conversion efficiency of 8.9% under reverse scanning, while the open-circuit voltage (Voc) increased from 0.55 to 0.63 V. Moreover, the FASnI_3_–PVA devices demonstrated long-term operational stability, maintaining their initial efficiency without noticeable degradation after 400 h of maximum-power-point operation. This method, based on O–H…I^−^ hydrogen-bond interactions between PVA and FASnI_3_, can be broadly applied to enhance both the efficiency and stability of FASnI_3_-based perovskite solar cells.

As demonstrated in previous studies, promising tin-based perovskite solar cells face significant limitations associated with the oxidation of Sn^2+^ to Sn^4+^, which leads to reduced power conversion efficiency and compromised operational stability. In the work [68], the authors employed phenylethylammonium bromide (PEABr) to form an ultrathin, low-dimensional perovskite layer on the surface of the FASnI_3_ absorber, thereby improving the perovskite/PCBM interface in the inverted device architecture ITO/PEDOT:PSS/perovskite/PCBM/BCP. The PEABr treatment increased the device efficiency from 4.77% to 7.86%. Experimental analyses revealed that the incorporation of PEABr promotes Br inclusion into the FASnI_3_ lattice and induces the formation of a thin low-dimensional interfacial layer, which enhances crystallinity, reduces defect density, suppresses Sn^2+^ oxidation, and improves band alignment. These effects collectively result in a pronounced improvement in power conversion efficiency.

Furthermore, the modified devices exhibit substantially enhanced stability: approximately 80% of the initial efficiency is retained after 350 h of continuous illumination, whereas the untreated control device degrades within 140 h.

In study [69], the authors demonstrated that the crystallization process of the FASnI_3_ perovskite is strongly governed by the conditions at the solution–air interface. To regulate this process, a specially designed additive—pentafluorophenoxyethylammonium iodide (FOEI)—was introduced into the perovskite precursor solution, effectively reducing the surface energy at this interface. This modification enables the formation of a highly oriented and smooth FASnI_3_–FOEI perovskite film with an extended charge-carrier lifetime, resulting in a certified power conversion efficiency of 10.16%.

The incorporation of N_2_H_5_Cl enables a reduction in the Sn^4+^ content in the FASnI_3_ film by approximately 20%, which in turn decreases charge-carrier recombination and facilitates the formation of a uniform, dense, pinhole-free perovskite layer. As a result of these improved structural properties, the FASnI_3_ film allows the device to reach a power conversion efficiency of 5.4%, primarily due to a notable enhancement in the open-circuit voltage. Furthermore, the best-performing PSC without encapsulation exhibits excellent storage stability, retaining about 65% of its initial efficiency after 1000 h [70].

The authors proposed an approach to enhance the efficiency and reproducibility of FASnI_3_-based PSCs by introducing the reducing agent LFA into the perovskite precursor solution. This modification enables the formation of highly crystalline FASnI_3_ films with lower Sn^4+^ content, reduced background doping, and decreased trap density. The resulting lead-free tin halide PSCs exhibit efficiencies exceeding 10%. It is anticipated that this reproducible strategy can be effectively applied to improve the performance of PSCs based on other tin-containing or mixed Sn–Pb perovskites used as active layers [71].

As noted, tin-based perovskites such as FASnI_3_ possess favorable optoelectronic properties, yet their films typically form with low quality. In this work [72], the addition of trimethylthiourea (3T) during spin coating improved the film morphology by promoting the merging of individual crystalline grains. This approach extended the charge-carrier lifetime to 123 ns and increased the open-circuit voltage to 0.92 V, resulting in a power conversion efficiency of 14% and improved resistance to moisture-induced degradation.

Wu and co-authors proposed an approach to reduce charge recombination by creating a built-in electric field inside the perovskite. To achieve this, an FASnI_3_ layer with a vertical Sn^2+^ gradient was fabricated using a Lewis base-assisted recrystallization method. This gradient strengthens the internal electric field and decreases recombination losses.

The analysis showed an upward shift in the Fermi level with increasing Sn^2+^ content, which helps prevent the trapping of charge carriers. In addition, the Sn^2+^-gradient perovskite absorber delivers an efficiency of 13.82% and a Voc enhancement of 130 mV, while the optimized device maintains over 13% efficiency after 1000 h of continuous operation under 1-sun illumination [73].

### 3.3. Cesium Tin Halides (CsSnX_3_)

Inorganic perovskites CsSnX_3_ were first synthesized and investigated by Scaife and colleagues; however, interest in these materials remained limited for quite some time [74]. The situation changed when Chen et al. demonstrated the application of CsSnI_3_ in perovskite solar cells in 2012 [75]. A black CsSnI_3_ film was produced by thermal annealing after sequential deposition of CsI and SnCl_2_ onto a glass substrate. The resulting CsSnI_3_-based solar cell exhibited a bandgap of 1.3 eV and a power conversion efficiency of 0.9%.

This study implements an approach based on pseudohalide anion alloying in CsSnI_3_ to regulate the crystallization of the inorganic perovskite and to form large micron-sized grains. The introduction of the pseudohalide anion modifies the phase-transition pathways through the formation of intermediate species, thereby slowing down the crystallization of CsSnI_3_. The incorporation of HCOO^−^ into the CsSnI_3_ lattice further enhances the oxidation resistance of Sn^2+^ due to the strong interaction between formate anions and Sn^2+^ cations. The fabricated planar CsSnI_3_ perovskite solar cell with an inverted architecture and an active area of 4.05 mm^2^ demonstrates a certified power conversion efficiency of 13.68% under AM 1.5 illumination (100 mW cm^−2^), ranking among the best reported for fully inorganic CsSnI_3_-based perovskite devices [76].

The black orthorhombic phase of CsSnI_3_ is a promising material for solar cells, but its efficiency is reduced due to the oxidation of Sn^2+^ to Sn^4+^. The addition of phthalimide (PTM) helps stabilize Sn^2+^ by forming coordination bonds and reducing defects, thereby improving the film quality. The modified solar cells on rigid and flexible substrates achieve efficiencies of 10.1% and 9.6%, respectively. After encapsulation, the devices show high stability, retaining 94.3% of their initial efficiency after 60 days in an inert atmosphere, 83.4% after 45 days in ambient air, and 81.3% after 2000 min of continuous 1-Sun illumination at approximately 70° [77].

In the following study [78], it is shown that air-stable solar cells based on B-γ CsSnI_3_ can be fabricated by introducing N,N′-methylenebis(acrylamide) (MBAA) into the perovskite layer and using poly(3-hexylthiophene) as the hole-transporting material. The lone electron pairs of the −NH and −CO functional groups in MBAA form coordination bonds with Sn^2+^, which reduces the concentration of Sn^4+^ defects and enhances the stability of the perovskite absorber under various conditions.

As a result of this modification, unencapsulated CsSnI_3_-MBAA solar cells achieve a power conversion efficiency of 7.50% when operated under ambient air. In addition, MBAA-treated devices retain 60.2%, 76.5%, and 58.4% of their initial efficiency after 1440 h of storage in an inert atmosphere, 120 h of exposure to ambient air, and 120 h of continuous 1-Sun illumination, respectively.

In this work [79], a co-additive—2-aminopyrazine (APZ)—is introduced into the precursor solution to form the SnF_2_·APZ complex, which effectively suppresses the oxidation of Sn^2+^ and thereby enhances the performance of CsSnI_3_-based devices. It is shown that the amino group in APZ significantly reduces Sn^2+^ oxidation through a Lewis acid–base interaction.

As a result, mesoporous perovskite solar cells based on CsSnI_3_, fabricated using a printable c-TiO_2_/m-TiO_2_/Al_2_O_3_/NiO/carbon architecture and exhibiting high reproducibility, achieve a power conversion efficiency of 5.12%. This result demonstrates the potential of fully inorganic mesoporous CsSnI_3_-based devices for photovoltaic applications. Moreover, after 60 days of storage in a nitrogen-filled glovebox, the device retained 92% of its initial efficiency.

CsSnI_3_ exhibits a sequence of temperature-dependent structural transformations among three black perovskite phases, denoted as B-α, B-β, and B-γ. As summarized in Table 5, cooling from elevated temperatures progressively lowers the lattice symmetry from cubic to tetragonal and subsequently to orthorhombic. These transitions are accompanied by increasing tilting and distortion of the SnI_6_ octahedra, which alter the Sn–I–Sn bond angles, orbital overlap, electronic band structure, and charge-carrier transport. The reported transition temperatures may vary slightly depending on sample preparation, crystallite size, and measurement conditions [80].

The cubic B-α phase is stable at elevated temperatures and exhibits an ideal three-dimensional network of corner-sharing SnI_6_ octahedra. Upon cooling, it transforms into the tetragonal B-β phase at approximately 425 K, followed by the formation of the orthorhombic B-γ phase below approximately 351 K. The reduction in symmetry is associated with a progressive increase in octahedral tilting and lattice distortion. Although the black B-γ phase is photoactive and can exist near room temperature, it is metastable under ambient conditions and may transform into the yellow non-perovskite Y phase in the presence of moisture and oxygen. This reconstructive degradation process is distinct from the reversible temperature-induced transitions among the black perovskite phases.

### 3.4. Vacancy-Ordered Sn(IV) Perovskites: (NH_4_)_2_SnCl_6_ and Cs_2_SnI_6_

Ammonium hexachlorostannate, (NH_4_)_2_SnCl_6_, is a vacancy-ordered Sn(IV) halide belonging to the A_2_BX_6_ structural family. It crystallizes in the cubic ($Fm\bar{3}m)$ space group with a lattice parameter of approximately 10.26 Å and four formula units per unit cell [80]. The Sn^4+^ ions occupy the crystallographic ((0,0,0)) positions and are coordinated by six Cl^−^ ions, forming isolated [SnCl_6_]^2−^ octahedra. The NH_4_^+^ cations are located at the ((1/4,1/4,1/4)) sites and provide charge compensation within the face-centered cubic lattice.

Unlike conventional ABX_3_ perovskites, in which the [BX_6_] octahedra form a continuous corner-sharing framework, the octahedra in (NH_4_)_2_SnCl_6_ are spatially isolated. This reduced structural connectivity affects orbital overlap, band dispersion, and charge-carrier transport. The presence of Sn^4+^ also distinguishes this compound from Sn^2+^-based perovskites, which are susceptible to oxidation and associated self-doping. Although (NH_4_)_2_SnCl_6_ has been investigated mainly as a model system for structural and dynamic studies and has not been established as an efficient photovoltaic absorber, it provides a useful structural reference for understanding vacancy-ordered Sn(IV) halides [81].

The potential relevance of (NH_4_)_2_SnCl_6_ to flexible photovoltaic materials was demonstrated in low-temperature SnO_2_-based composite films. Transparent films with a thickness of approximately 100–120 nm were deposited on flexible polyethylene terephthalate substrates by spray pyrolysis at 100 °C using aqueous–alcoholic SnCl_4_ solutions. The addition of aqueous ammonia promoted the formation of skeletal vacancy-ordered (NH_4_)_2_SnCl_6_ crystals within the SnO_2_ film, as confirmed by X-ray diffraction. Their incorporation reduced the bandgap of the film material from 4.04 to 2.64 eV while preserving its transparency and flexibility. These findings indicate that SnO_2_/(NH_4_)_2_SnCl_6_ composite films may be suitable for further investigation as functional layers in flexible solar cells. However, no complete photovoltaic devices or PCE measurements were reported in this study [82].

The photovoltaic relevance of this structural family is more clearly demonstrated by Cs_2_SnI_6_. This compound retains the vacancy-ordered A_2_SnX_6_ framework but contains Cs^+^ and I^−^ instead of NH_4_^+^ and Cl^−^, respectively. The substitution of chloride with iodide increases the polarizability of the anionic framework and shifts the optical response toward the visible and near-infrared regions. Consequently, Cs_2_SnI_6_ exhibits a considerably narrower bandgap and stronger visible-light absorption than (NH_4_)_2_SnCl_6_, making it more suitable for photovoltaic applications. Therefore, the following discussion focuses on the structural, optoelectronic, and device-related properties of Cs_2_SnI_6_.

#### Cesium Tin Iodide, Cs_2_SnI_6_

Cs_2_SnI_6_ belongs to the family of vacancy-ordered double perovskites and exhibits relatively high stability under ambient conditions. This stability is associated with the presence of tin predominantly in the Sn^4+^ oxidation state, which reduces the susceptibility of the material to further oxidation. In addition to its environmental stability, Cs_2_SnI_6_ exhibits strong optical absorption and a tunable bandgap, making it a potential lead-free material for photovoltaic applications. Depending on its composition, defect chemistry, and preparation conditions, Cs_2_SnI_6_ can exhibit either p-type or n-type semiconducting behavior [83].

Initial studies employed Cs_2_SnI_6_ primarily as a p-type semiconductor and hole-transport material in dye-sensitized solar cells (DSSCs) [84]. It was subsequently investigated as an active component in perovskite photovoltaic devices, although the reported efficiencies remained below 10% [85]. The potential use of Cs_2_SnI_6_ as either a charge-transport or light-absorbing layer is supported by its optical bandgap, which can be tuned within the range of 1.25–1.62 eV. This interval includes values close to the Shockley–Queisser optimum of approximately 1.34 eV for single-junction solar cells. The combination of suitable optoelectronic properties and ambient stability therefore supports the further development of Cs_2_SnI_6_ for stable lead-free photovoltaic devices [85].

A rapid mechanochemical route was developed for synthesizing Cs_2_SnI_6_ through the reaction of pre-prepared CsI and SnI_4_ in the presence of hydroiodic acid. The synthesis was completed within 12 min under mild conditions. Rietveld refinement confirmed that the resulting Cs_2_SnI_6_ retained its crystallinity and phase purity for more than four months under ambient conditions at a relative humidity exceeding 70%. When incorporated into a dye-sensitized solar cell, Cs_2_SnI_6_ increased the power conversion efficiency by 32% relative to the control device, which was attributed to enhanced light absorption and favorable energy-level alignment. Integration of the Cs_2_SnI_6_-based DSSC with a supercapacitor enabled photocharging and provided a discharge specific capacitance of 92.12 F g^−1^ at 1 A g^−1^. The integrated device retained 81.27% of its initial performance after 30 cycles under one-sun illumination and showed at least a 60% improvement in stability under halogen-lamp illumination, even without encapsulation. These results demonstrate the potential of mechanochemically synthesized Cs_2_SnI_6_ for integrated photovoltaic energy-harvesting and storage devices, including indoor-light applications [86].

Zaman et al. conducted a computational study using SCAPS-1D to optimize the architecture of an all-inorganic Cs_2_SnI_6_-based solar cell. Seven electron-transport layers and six hole-transport layers were comparatively evaluated, with WS_2_ and SrCu_2_O identified as the most compatible ETL and HTL, respectively. The effects of absorber thickness, bandgap, bulk and interfacial defect densities, donor and acceptor concentrations, operating temperature, and back-contact material were systematically investigated. Under optimized simulation conditions, the FTO/WS_2_/Cs_2_SnI_6_/SrCu_2_O/Ni device exhibited a (*V*_OC_) of 1.06 V, a (*J*_SC_) of 30.82 mA cm^−2^, an FF of 87.11%, and a calculated PCE of 28.43%. These results represent a theoretical performance prediction rather than an experimentally measured efficiency and provide design guidelines for the future development of Cs_2_SnI_6_ photovoltaic devices [87].

### 3.5. Two-Dimensional Dion-Jacobson Tin Halide Perovskites

Layered two-dimensional (2D) perovskites have attracted considerable interest because they generally exhibit improved structural and environmental stability compared with their three-dimensional counterparts [88]. The reduced dimensionality is achieved by introducing bulky organic spacer cations between the inorganic metal-halide slabs. Depending on the charge and molecular structure of these spacers, layered perovskites are commonly classified into Ruddlesden–Popper (RP) and Dion–Jacobson (DJ) phases.

The RP family is described by the general formula A′_2_A_n−1_B_n_X_3n+1_, where A′ represents a bulky monovalent organic spacer and (*n*) denotes the number of inorganic octahedral layers between adjacent organic layers. RP perovskites were first incorporated into solar cells by introducing phenylethylammonium (PEA^+^) into MAPbI_3_, resulting in the formation of the layered compound (PEA)_2_(MA)_2_Pb_3_I_10_ with (*n* = 3) [89]. Although the corresponding devices exhibited a PCE of 4.7%, the perovskite films showed improved stability at room temperature under an ambient relative humidity of 52% [89]. Subsequent progress has been driven by advances in solution processing, spacer-cation design, crystallization control, and a better understanding of the physical properties of layered perovskite films.

**Figure 4:**
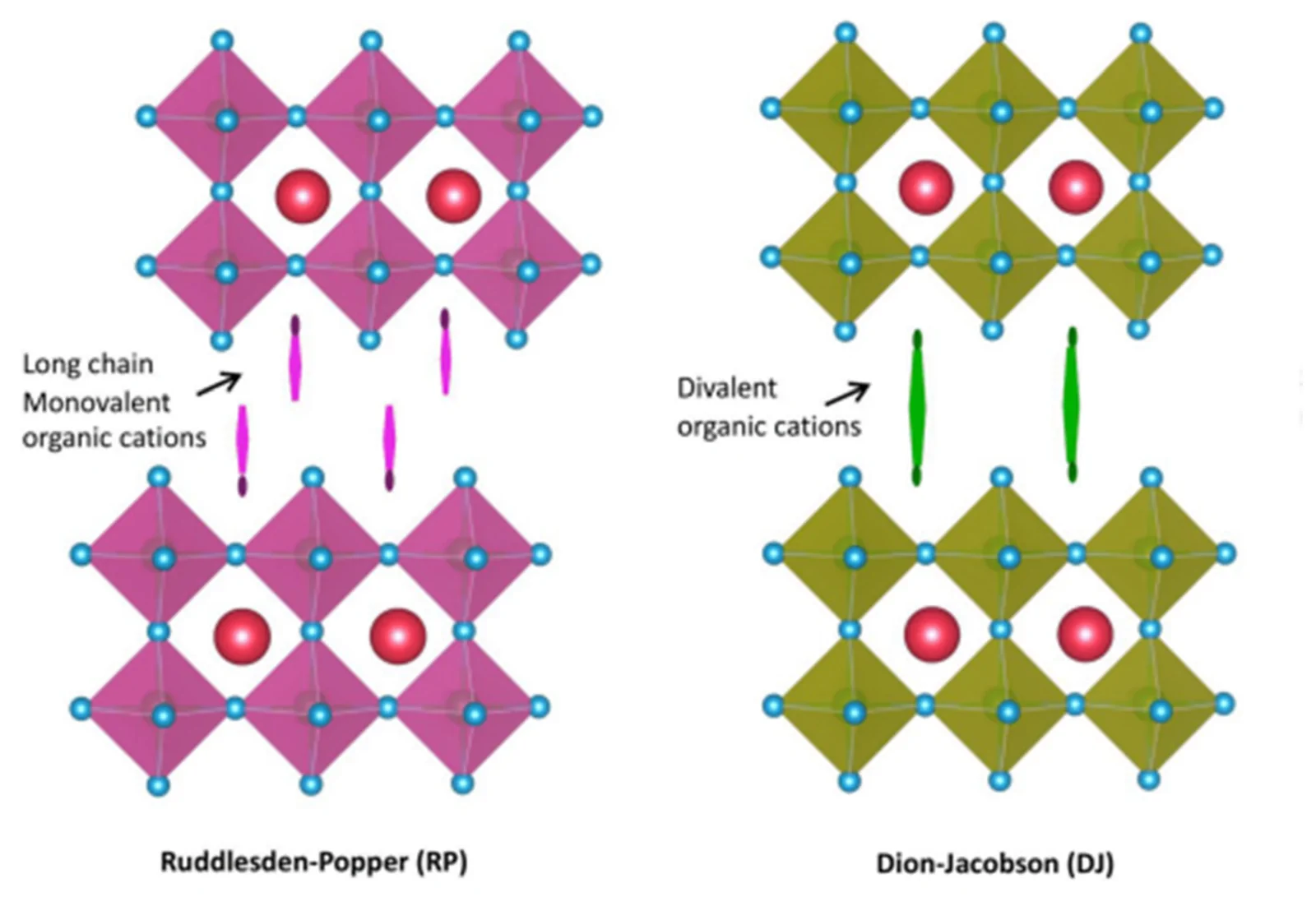
Schematic illustration of the Ruddlesden–Popper (RP) and Dion–Jacobson (DJ) phases of two-dimensional perovskites. Reproduced from Ref. [92] under the Creative Commons Attribution 4.0 International License (CC BY 4.0).

In contrast to RP perovskites, which contain two monovalent spacer layers separated by van der Waals interactions, DJ perovskites incorporate divalent diammonium cations that bridge adjacent metal-halide slabs. Hydrogen bonding between both ammonium termini and the inorganic layers strengthens the interlayer interaction and eliminates the van der Waals gap. This structural arrangement contributes to the mechanical rigidity and structural stability of the DJ framework [90,91]. The principal structural differences between RP and DJ layered perovskites are illustrated in Figure 4.

The emergence of two-dimensional (2D) halide perovskites and their integration into hierarchical 2D/3D structures has become an important step toward developing highly efficient and stable solar cells. Among them, Dion–Jacobson (DJ) phase perovskites are of particular interest due to their improved charge-transport properties. Ke and co-authors described 2D DJ-type perovskites formed using the organic spacer 3-(aminomethyl)piperidinium (3AMP), which enable the fabrication of mixed Pb/Sn perovskites with a narrow bandgap of 1.27 eV and a long carrier lifetime of 657.7 ns.

The environmental stability of lead-free Dion–Jacobson tin halide perovskites has also been investigated using the bromide-based compound HDASnBr_4_, where HDA denotes hexane-1,6-diammonium. Exposure to ambient humidity altered the relative fractions of the two-dimensional HDASnBr_4_ phase and the hydrated one-dimensional HDA_3_SnBr_8_ phase. During the first approximately 40 h of exposure, the luminescence intensity increased because of the growing contribution of the hydrated 1D phase, which promotes self-trapped exciton emission. Prolonged exposure subsequently caused material degradation and a reduction in luminescence. The addition of SnCl_2_ and tetraethyl orthosilicate extended the luminescence lifetime of the thin films to more than 260 h, compared with approximately 180 h for films without additives. However, the improved persistence of luminescence was attributed primarily to changes in the evolution of the 2D and hydrated 1D phase fractions rather than exclusively to the suppression of Sn^2+^ oxidation [93].

Another promising strategy involves the development of two-dimensional Dion–Jacobson (DJ) tin-based perovskites stabilized by diammonium organic spacers. In this regard, three phenylenediammonium (PDA) isomers, namely ortho- (o-PDA), meta- (m-PDA), and para- (p-PDA), were systematically investigated. Among them, o-PDA exhibited the most favorable structural and optoelectronic characteristics. Its relatively large molecular dipole moment reduced the exciton binding energy, while the formation of strong hydrogen bonds between the o-PDA cation and the inorganic Sn–I octahedral framework significantly enhanced the structural stability of the perovskite. Furthermore, the incorporation of o-PDA promoted the formation of compact and highly uniform thin films while simultaneously improving the antioxidant protection of the perovskite, thereby contributing to enhanced device stability [94]. Femtosecond transient absorption spectroscopy further revealed that o-PDA reduced the fraction of undesirable small-n phases, thereby facilitating more efficient charge transfer between layers with different n-values. Consequently, solar cells based on the 2D Dion–Jacobson (o-PDA)FA_3_Sn_4_I_13_ perovskite achieved a power conversion efficiency of 7.18%.

Organic spacer cations in 2D perovskites create strong dielectric confinement and quantum-well effects, which weaken charge transport and reduce the efficiency of solar cells. In the following study, the authors propose replacing the conventional p-phenylenediammonium with a larger aromatic spacer, 1,5-naphthalenediammonium (15-NDA). Theoretical calculations and experimental results show that the extended π-system of 15-NDA introduces greater steric hindrance and suppresses the formation of small n-phases. This reduces dielectric and quantum confinement, improving exciton dissociation. The strong π–π interactions of 15-NDA also promote the formation of dense, uniform films and decrease the oxidation of Sn^2+^ and I^−^, lowering the defect density.

As a result, the devices exhibited longer carrier diffusion lengths and more efficient charge extraction. Solar cells based on the 2D Dion–Jacobson (1,5-NDA)FA_3_Sn_4_I_13_ perovskite achieved a power conversion efficiency of 7.33% and demonstrated improved stability under humid conditions and in a nitrogen atmosphere [95].

### 3.6. Two-Dimensional Ruddlesden–Popper (RP) Tin Halide Perovskites

Two-dimensional Ruddlesden–Popper (RP) tin halide perovskites generally exhibit superior environmental stability compared with their three-dimensional counterparts. However, their photovoltaic performance remains limited by the complex and difficult-to-control crystallization process, which often results in incomplete preferential crystal orientation and poor film quality. To address this challenge, the authors introduced a mixed organic spacer strategy by combining n-butylammonium (BA) and phenethylammonium (PEA) cations. In the mixed-spacer perovskite, [(BA_0.5_PEA_0.5_)_2_FA_3_Sn_4_I_13_], the synergistic effect of BA^+^ and PEA^+^ effectively suppressed the formation of intermediate phases that hinder homogeneous nucleation and oriented crystal growth. As a result, the perovskite films exhibited improved morphology, enhanced crystallographic orientation, and superior structural quality. Consequently, the corresponding two-dimensional Ruddlesden–Popper tin-based perovskite solar cells achieved a power conversion efficiency of 8.82% [96].

Yin et al. proposed a strategy for fabricating quasi-two-dimensional (quasi-2D) tin perovskite films by combining Dion–Jacobson (DJ) and Ruddlesden–Popper (RP) phase cations to construct a mixed-phase perovskite (MPP) structure. The resulting quasi-2D MPP films with the composition EDA_0.7_(EA_2_)_0.3_FA_9_Sn_10_I_31_ (where EDA denotes ethylenediammonium and EA represents ethylammonium) exhibited reduced defect density, enhanced crystallinity, and improved hydrophobicity compared with their pure RP- and DJ-phase counterparts. These structural improvements translated into enhanced photovoltaic performance, enabling quasi-2D MPP tin perovskite solar cells to achieve a power conversion efficiency (PCE) of 7.20%. Furthermore, the unencapsulated devices demonstrated remarkable operational stability, retaining 83% of their initial PCE after 2000 h of storage under a nitrogen atmosphere and more than 80% after 200 h of exposure to ambient air with a relative humidity of 45–55% [97].

Zibouche et al. investigated the structural and electronic properties of two-dimensional Ruddlesden–Popper (RP) lead- and tin-based perovskites with different numbers of inorganic layers (*n* = 1–4). Their calculations revealed that the bandgap increases with decreasing layer number in both Sn- and Pb-based systems, reflecting the enhanced quantum confinement effect in low-dimensional structures. Substituting methylammonium (MA) with formamidinium (FA) produced different electronic responses depending on the metal cation: a slight increase in the bandgap was predicted for tin-based perovskites, whereas a reduction was observed for their lead-based counterparts.

The calculated electron and hole effective masses remained relatively low in all investigated 2D structures, indicating high charge-carrier mobility, in some cases exceeding that of the corresponding three-dimensional perovskites. Furthermore, the orientation of the organic MA and FA cations induced structural distortions that gave rise to Rashba-type band splitting in multilayer RP perovskites.

Band alignment analysis further revealed a directional carrier-transport mechanism, in which electrons preferentially migrate from regions with smaller n values toward thicker perovskite slabs with larger n, while holes exhibit the opposite transport direction. These theoretical predictions are consistent with experimental observations. In addition, the authors suggested that RP two-dimensional perovskites can function as efficient hole-transporting interlayers when integrated with three-dimensional perovskite absorbers, highlighting their potential for improving charge extraction and device performance [98].

Collectively, these studies demonstrate that precise control over crystallization and phase engineering is the key to improving both the efficiency and stability of two-dimensional tin-based perovskites. Strategies such as the incorporation of mixed organic spacers (BA/PEA), the combination of Dion–Jacobson (DJ) and Ruddlesden–Popper (RP) phase cations, and a deeper understanding of the electronic properties of multilayer RP structures enable the fabrication of perovskite films with improved morphology, reduced defect density, and enhanced crystallographic orientation. These advances have led to significant improvements in photovoltaic performance, with power conversion efficiencies reaching 8.82% and 7.20%, together with substantially enhanced device stability. Furthermore, theoretical investigations have confirmed the intrinsically high charge-carrier mobility and directional charge transport in multilayer RP perovskites, highlighting their potential not only as efficient hole-transporting materials but also as promising building blocks for the development of highly stable lead-free perovskite solar cells.

To systematize the current progress in the field of lead-free tin-based perovskite solar cells, a comparative assessment of the most significant studies of recent years has been carried out. Table 6 includes both experimental investigations aimed at suppressing Sn^2+^ oxidation, controlling crystallization, and passivating defects, as well as numerical models employing SCAPS-1D, machine learning, and optical–electrical simulations to optimize device architecture.

Chen et al. introduced cis- and trans-isomeric pyridyl-substituted fulleropyrrolidines, denoted CPPF and TPPF, respectively, as precursor additives for PEA/FA tin iodide perovskite solar cells. Owing to the larger spatial separation between its pyridine groups, TPPF interacted with multiple perovskite colloids through coordination bonds, slowed the crystallization process, and reduced excessive nucleation. This resulted in films with improved crystallographic orientation, compactness, and interfacial energy-level alignment, while simultaneously suppressing Sn^2+^ oxidation. The optimized inverted device with an ITO/PEDOT:PSS/perovskite/PCBM/BCP/Ag architecture achieved a PCE of 15.38%, with a *V*oc of 0.856 V, a *J*sc of 24.81 mA cm^−2^, and an FF of 72.37%. An independently certified PCE of 15.14% was obtained. Furthermore, the unencapsulated devices retained approximately 99% of their initial efficiency after 3000 h of storage and 93% after 500 h of continuous illumination. These results demonstrate that controlling precursor coordination can simultaneously regulate crystallization, reduce Sn^2+^ oxidation, and improve the efficiency and stability of tin-based perovskite solar cells [99].

More recent progress has been achieved through electron-transport-layer engineering in fullerene-free tin-based perovskite solar cells. Li et al. replaced the conventional fullerene-based electron-transport layer with fluorinated triple-acceptor polymers (P1–P3) in inverted PEA_0.10_FA_0.90_SnI_3_ devices. Among these materials, the P3 polymer provided favourable energy-level alignment, high electron mobility, uniform interfacial coverage and strong interactions with the perovskite surface. These properties facilitated electron extraction, reduced the interfacial defect density and suppressed non-radiative recombination. The optimized 0.04 cm^2^ device achieved a PCE of 16.06%, with a *V*oc of 0.99 V, a *J*sc of 19.79 mA cm^−2^ and an FF of 81.98%; an independently certified efficiency of 15.90% was obtained. When the active area was increased to 1 cm^2^, the device exhibited a PCE of 14.67%, with a *V*oc of 0.98 V, a *J*sc of 19.72 mA cm^−2^ and an FF of 76.47%, while the certified efficiency reached 14.51%. The P3-based devices also exhibited improved operational stability, which was attributed to the hydrophobic long-alkyl side chains, fluorine substituents and strong interfacial interactions of the polymer ETL [100].

**Table 6:** Sn-perovskite material and solar cell efficiencies.

Perovskites	Year	Method	Important Feature/Performance Enhancement Strategy	Band Gap (eV)	*J*sc [mA/cm^2^]	FF [%]	*V*oc [V]	PCE [%]	Ref
MASnI_2_Br	2026	SCAPS simulation	Optimized ETL/HTL	1.56	24.4	82.6	1.17	23.6	[101]
MASnIBr_2_	2025	SCAPS-1D simulation;	optimized HTL/ETL design	1.75	17.91	82.75	1.38	20.42	[102]
MASnI_3_	2021	SCAPS-1D simulation	Optimization of ETM, HTM and back metal contacts (PCBM/CuI/Au)	1.30	34.27	69.23	1.056	25.05	[103]
MASnI_3_	2019	Experimental	Cation-exchange approach; hydrazinium-assisted fabrication; suppressed Sn^2+^ oxidation	~1.30	22.91	64	0.486	7.13	[61]
MAPbI_3_/MASnI_3_ (half tandem)	2025	Optical–electrical simulation (FEM)	Half-tandem architecture with TiO_2_@Ag@TiO_2_/SiO_2_@Ag@SiO_2_ triple core–shell plasmonic nanoparticles	1.55/1.30	35.17	84.16	1.01	30.18	[62]
MASnI_3_	2026	SCAPS-1D simulation + Machine learning	WS_2_ ETL + Zn_3_P_2_ HTL optimization; ML-assisted parameter optimization	1.30	34.19	87.35	1.08	32.30	[104]
MASnI_3_	2017	Experimental	Modified solvent bathing method for high-coverage perovskite films	-	11.82	40	0.45	2.14	[105]
MASnI_3_	2024	SCAPS-1D simulation	Graphene and ZnO:Al interface engineering to suppress recombination losses	1.30	29.40	89.30	1.159	30.43	[106]
FASnI_3_–PVA	2019	Experimental	Hydrogen-bond engineering (PVA additive) for crystallization control and defect passivation	1.37	20.37	69.3	0.632	8.92	[67]
FASnI_3_	2019	Experimental	PEABr interface engineering	1.40	22.64	64.0	0.54	7.86	[68]
FASnI_3_–FOEI	2020	Experimental	Surface-controlled oriented crystal growth using FOEI additive (surface-energy engineering);	1.34	21.59	75.0	0.67	10.81	[69]
FASnI_3_	2018	Experimental	Hydrazinium chloride (N_2_H_5_Cl) coadditive engineering; suppression of Sn^2+^ oxidation and pinhole-free film formation	1.37	17.64	67.3	0.455	5.40	[70]
FASnI_3_ + 50% LFA	2020	Experimental	Volatilizable liquid formic acid (LFA) reducing solvent; suppression of Sn^2+^ oxidation and reduced trap density	1.37	22.08	77.0	0.61	10.37	[71]
FASnI_3_	2022	Experimental	Trimethylthiourea (3T) ligand engineering for crystallization control and surface passivation; smooth and compact FASnI_3_ films	1.40	20.4	76.7	0.92	14.0	[72]
FASnI_3_	2022	Experimental	Lewis base-assisted recrystallization with PMMA; vertical Sn^2+^ gradient and enhanced built-in electric field	1.41	22.74	71.5	0.85	13.82	[73]
CsSnI_3_	2024	Experimental	Pseudohalide anion alloying (Cs formate, HCOO^−^); crystallization control and Sn^2+^ oxidation suppression	1.30	24.94	74.0	0.75	13.68 (certified)	[76]
B-γ CsSnI_3_	2021	Experimental	Localized electron density engineering using phthalimide (PTM); trigeminal coordination to suppress Sn^2+^ oxidation and defect passivation	1.31	21.81	72.1	0.64	10.10	[77]
PEA/FA tin iodide perovskite (PEAI:FAI:SnI_2_ = 0.15:0.85:1; SnF_2_ additive)	2024	Experimental, supported by DFT calculations	Trans-isomeric fulleropyrrolidine (TPPF) precursor additive regulates crystallization, improves film orientation and compactness, optimizes interfacial energy-level alignment, and suppresses Sn^2+^ oxidation	-	24.81	72.37	0.856	15.38 (certified: 15.14)	[99]
PEA_0.10_FA_0.90_SnI_3_ (0.04 cm^2^)	2026	Experimental, supported by DFT calculations	Fullerene-free fluorinated triple-acceptor polymer P3 used as the ETL; improved energy-level alignment, electron mobility, interfacial contact and defect passivation; suppressed interfacial recombination	1.39	19.79	81.98	0.99	16.06 (certified: 15.90)	[100]
PEA_0.10_FA_0.90_SnI_3_ (1 cm^2^)	2026	Experimental, supported by DFT calculations	Fullerene-free P3 ETL with uniform conformal coverage, efficient electron extraction, strong interfacial interaction and improved scalability	1.39	19.72	76.47	0.98	14.67 (certified: 14.51)	[100]
MASnI_3_ (FTO/ZnSe/MASnI_3_/NiO)	2025	SCAPS-1D simulation	Optimized ETL (ZnSe) and HTL (NiO); absorber thickness 920 nm; reduced defect densities	~1.2–1.4	33.64	81.22	1.088	29.75	[107]

The consolidated table brings together data on various Sn-perovskite compositions (MASnX_3_, FASnI_3_, CsSnI_3_ and their derivatives), fabrication methods, strategies for improving stability and efficiency, and key photovoltaic parameters—bandgap, short-circuit current density, fill factor, open-circuit voltage, and power conversion efficiency.

This overview provides a clear illustration of how approaches to improving Sn-based perovskites have evolved: from interface engineering and additive-assisted crystallization control to the development of new charge-transport layers and optimization of device architecture.

## 4. Conclusions and Perspectives

Tin-based perovskite solar cells have made notable progress over the past decade, yet their advancement toward practical and commercially viable photovoltaic technologies remains constrained by several fundamental challenges. The intrinsic chemical instability of Sn^2+^, which readily oxidizes to Sn^4+^ even under mild environmental conditions, continues to be the primary obstacle. This oxidation process leads to elevated background carrier concentrations, increased defect densities, enhanced non-radiative recombination, and accelerated structural degradation, ultimately limiting device efficiency and operational lifetime. Consequently, tin-based perovskite devices still exhibit lower open-circuit voltages and reduced stability compared with their lead-based counterparts.

Equally significant are the issues associated with uncontrolled crystallization and film formation. Rapid nucleation and growth of tin halide perovskites often result in non-uniform films with pinholes and high trap densities, which severely hinder charge transport and increase recombination losses. Interface instability between the perovskite absorber and charge-transport layers further contributes to performance degradation, underscoring the need for improved interfacial engineering.

Despite these limitations, tin-based perovskites remain highly promising materials for next-generation photovoltaics. Their narrow band gaps, strong optical absorption, and compatibility with tandem architectures position them as attractive candidates for high-efficiency, lead-free solar cells. Recent advances in precursor chemistry, reducing additives, 2D/3D heterostructures, and interface modification demonstrate that many intrinsic drawbacks can be mitigated through rational materials design. The integration of machine learning and high-throughput computational screening is expected to accelerate the discovery of new tin-based compositions with enhanced stability and optimized optoelectronic properties.

Looking forward, the future success of tin-based perovskites will depend on the development of robust strategies to stabilize the Sn^2+^ oxidation state, suppress defect formation, and improve long-term environmental durability. Equally important is the adaptation of scalable fabrication methods—such as blade coating, slot-die coating, and vapor deposition—to accommodate the rapid crystallization kinetics of tin halides. If these challenges are effectively addressed, tin-based perovskites may evolve into a cornerstone of sustainable, high-efficiency photovoltaic technologies, offering a viable alternative to lead-containing materials.

This review is expected to provide valuable guidance for future research on tin-based perovskite materials by deepening the understanding of their coordination chemistry, phase behavior, defect formation pathways, and interface engineering strategies, thereby supporting the development of multifunctional lead-free systems for sustainable energy applications.

## Figures and Tables

**Table 1:** Physical and chemical properties of Sn relevant to photovoltaic applications.

Property	Significance	Ref
Atomic number	50	
Atomic mass	118.710 g/mol	
Density (white tin)	7280 g cm^−3^	[19]
Density (grey tin)	5769 g/cm^−3^	
Melting point	231.9 °C	
Boiling point	2620 °C	
Standard enthalpy of formation	0 kJ/mol (elemental Sn, standard state)	
Melting heat	7.03 kJ mol^−1^ (≈59.2 J g^−1^)	

**Table 2:** Estimated world mine production of tin by country in2024 and 2025 [37,38].

Country	Mine Production		Reserves
	2024	2025	
United States	—	—	—
Australia	11,300	12,000	570,000
Bolivia	21,200	15,000	400,000
Brazil	27,600	28,000	700,000
Burma	20,000	12,000	700,000
China	71,000	71,000	1,200,000
Congo (Kinshasa)	26,000	27,000	91,000
Indonesia	55,000	61,000	1,400,000
Laos	1860	1800	NA
Malaysia	5460	5000	NA
Nigeria	3100	3500	NA
Peru	32,300	33,000	150,000
Russia	3260	4500	460,000
Rwanda	4100	4600	NA
Vietnam	11,000	11,000	23,000
Other countries	1570	1700	310,000
World total (rounded)	294,000	290,000	>6,000,000

**Table 3:** Temperature-dependent phase sequence and structural characteristics of MASnI_3_ [59].

Phase	Crystal System	Space Group	Transition Temperature	Key Characteristics
α phase	Pseudocubic	$Pm\bar{3}m$	~275 K	High-symmetry, dynamically averaged perovskite structure; substantial orientational disorder of MA^+^ cations and dynamic motion of the SnI_6_ framework
β phase	Tetragonal	*I4/mcm*	Present between ~108 and 275 K upon cooling	Increased SnI_6_ octahedral tilting and reduced lattice symmetry; intermediate degree of MA^+^ orientational ordering
γ phase	Orthorhombic	*Pnma*	Forms below ~108 K upon cooling; reverse transition occurs near 114 K upon heating	Pronounced octahedral tilting and lattice distortion; increased ordering of MA^+^ cations; reduced defect-related disorder and longer charge-carrier lifetime

**Table 4:** Temperature-dependent phase transitions, crystal symmetry, and structural characteristics of FASnI_3_ [66].

Phase	Crystal System	Space Group	Transition Temperature	Key Characteristics
α phase	Cubic	$Pm\bar{3}m$	Stable near room temperature; cubic-to-tetragonal transition occurs between ~250 and 225 K	High-symmetry framework; dynamically disordered FA^+^ cations; reduced octahedral tilting
β phase	Tetragonal	*P4/mbm*	Stable at ~225 and 150 K	In-plane rotation of SnI_6_ octahedra; reduced lattice symmetry; partial orientational ordering of FA^+^ cations
γ phase	Orthorhombic	*Pnma*	~150 and 125 K and remains stable at lower temperatures	Pronounced octahedral tilting and lattice distortion; increased orientational ordering of FA^+^ cations

**Table 5:** Temperature-dependent phase transitions, crystal symmetry, and structural characteristics of CsSnX_3_ [80].

Phase	Crystal System	Space Group	Transition Temperature	Key Characteristics
B-α (Black-alpha)	Cubic	$Pm\bar{3}m$	Stable above ~425 K	Ideal 3D perovskite cage, highest symmetry.
B-β (Black-beta)	Tetragonal	*P4/mbm*	Stable between ~351 K and 425 K	Moderate distortion, intermediate cooling phase.
B-γ (Black-gamma)	Orthorhombic	*Pnma*	Stable at room temp < 351 K	Distorted 3D perovskite, direct bandgap (~1.3 eV).

## Data Availability

No new data were created or analyzed in this study.

## Abbreviations

The following abbreviations are used in this manuscript:

MA	Methylammonium [CH_3_NH_3_]^+^)
PCE	Power conversion efficiency
PSC	Perovskite solar cell
*V*oc	Open-circuit voltage
